# Albuminuria in the Apparently Healthy

**Published:** 1904-04-23

**Authors:** 


### ALBUMINURIA IN THE APPARENTLY HEALTHY.

Dr. Samuel West,[1] discussing this subject at the West London Medico-Chirurgical Society, spoke of the difficulty of assuring oneself that a patient who is apparently healthy is actually so, except for the passage of albumen in the urine. Albuminuria is rare in health. If a large quantity of normal urine be evaporated, concentrated, and the residue dissolved, a tiny trace of albumen may be demonstrated by delicate tests. This is the only true physiological albuminuria. In the strict sense of the term, albuminuria is never physiological, always pathological, though the person may be apparently healthy.

Causes of albuminuria fall into three groups:—(1) The accidental, normally secreted urine contaminated on its way out; (2) the renal, where there is more or less obvious cause in the kidney itself; (3) the pre-renal group, in which the cause is some general or local pathological condition as morbus cordis fever, or blood disorders. Functional albuminuria may be described as albuminuria in which the cause is not obvious. The diagnosis has to be made by exclusion. Accidental contamination is combated by the use of a catheter, but transient albuminuria may be caused by oxalates or uric acid gravel in the pelvis of the kidney. In the pre-renal group it is probable that toxins absorbed from the intestinal tract or generated within the body itself, play an important part. Transient albuminuria may follow the ingestion of certain articles of food, and appears to be due to elimination of a poison. Occasional, intermittent, remittent, cyclical, postural and dietetic albuminuria are names given to this form of urinary complaint, but the conditions to which these terms refer may be met with in convalescence from acute nephritis, and therefore are no proof that the kidneys are sound.

Functional albuminuria frequently occurs in infants and has little significance. Boys at school show it in a proportion of one in five. There are two classes, the pale and the florid, probably presenting differences in pathology, prognosis and treatment. As a rule the boy's health does not suffer if he is left at school, and the symptom eventually disappears. In the young adult there is a risk that the albuminuria may not clear up, so that an insurance office is justified in declining to take the risk, but as most of these cases recover entirely it seems unfair that lads so suffering should be debarred from appointments in business houses and banks. At the age period of 25 to 30 frequency of albuminuria is at its lowest. After the age of 30 it rises with every year of life, and with that rise the gravity also increases.

In granular kidney all the features of functional albuminuria may be reproduced; the albumen may be small in amount, not be always present, and vary with posture and diet. But if with albuminuria there be associated arterial thickening, there must be grave arterial or kidney disease, and the patient is not suffering from functional albuminuria alone. Insurance companies should refuse all cases of albuminuria over 40 years of age, load heavily those between 30 and 40, make an addition between 25 and 30, postpone and watch all between 18 and 25, reject at every age those who also have arterial thickening. Dr. West summed up by stating his conviction that albuminuria is never truly physiological, though it may be due to other than renal causes.

[1] West London Med. Jour., April, 1904.

